# MRI based volumetric lung nodule assessment - a comparison to computed tomography

**DOI:** 10.3389/fmed.2025.1491960

**Published:** 2025-04-03

**Authors:** Tatjana Dell, Andreas Feisst, Olga Ramig, Yannik Layer, Narine Mesropyan, Alexander Isaak, Claus Pieper, Patrick Kupczyk, Julian Luetkens, Daniel Thomas, Daniel Kuetting

**Affiliations:** ^1^Department of Diagnostic and Interventional Radiology, Quantitative Imaging Lab Bonn, University Hospital Bonn, Bonn, Germany; ^2^Department of Diagnostic and Interventional Radiology, St. Vinzenz Hospital, Cologne, Germany

**Keywords:** pulmonary nodules, lung cancer screening, magnetic resonance imaging, volumetry, early detection of cancer, low-dose computed tomography, radiation protection

## Abstract

**Purpose:**

Previous studies have demonstrated that nodule volumetry allows for the deduction of imaging-based biomarkers such as volume doubling time, enabling superior discrimination between benign and malignant lesions compared to 2D-based morphological characteristics. The study aimed to assess the feasibility and accuracy of *in-vivo* magnetic resonance imaging (MRI)-based volumetric assessment of lung nodules larger than 6 mm, in comparison to the current gold standard, CT.

**Materials and methods:**

This study involved a subgroup analysis of 233 participants from a prospective, single-center lung cancer screening program using CT and MRI. Patients were included if foci ≥6 mm were detected in CT during the initial screening round, resulting in 23 participants with 47 pulmonary nodules. MRI was performed using a 1.5 Tesla unit with a transverse T2-weighted MultiVane XD imaging technique, while low-dose CT (LDCT) was performed on a 128-slice spiral CT scanner. Volumetric nodule assessment was conducted using a computer-aided diagnosis system, with images reviewed by two experienced radiologists. Statistical analysis included regression analysis, Bland-Altman analysis, and calculation of the interclass correlation coefficient (ICC) to assess correlation and reproducibility.

**Results:**

Comparison of MRI-based volumetric assessment with LDCT as the reference standard revealed a mean nodule volume of 1.1343 ± 3.1204 cm^3^ for MRI versus 1.2197 ± 3.496 cm^3^ for LDCT (*p* = 0.203). Regression analysis demonstrated a strong linear relationship between the modalities (*r*^2^ = 0.981, *p* < 0.001), consistently observed even for nodules <5 cm^3^ (*r*^2^ = 0.755, *p* < 0.001). Bland-Altman analysis indicated no significant systematic bias in nodule volume measurements between MRI and CT, with a mean difference of 0.12 cm^3^ and narrow 95% confidence intervals (−6.852 to 6.854 cm^3^). Intra-reader reproducibility for CT-based volumetry was excellent (ICC = 0.9984), while MRI-based measurements showed good reproducibility (ICC = 0.7737). Inter-reader reproducibility was high for CT (ICC = 0.995) and moderate for MRI (ICC = 0.7135).

**Conclusion:**

This study demonstrates that MRI-based volumetry of lung nodules ≥6 mm is feasible and accurate, showing comparable precision to CT with minimal bias in volume measurements, and highlights the potential of MRI as a radiation-free alternative for lung nodule follow-up and screening.

## Introduction

Pulmonary nodules are detected in more than 1.5 million patients per year in the US and can be found in more than a third of all computed tomographic (CT) scans of the chest. These nodules frequently require follow up or other dedicated work up (1).

Volumetric lung nodule assessment is a more effective predictor of malignancy than two-dimensional (2D) evaluation based on diametric measurements. Nodule volumetry enables the deduction of imaging-based biomarkers, such as volume doubling time (VDT), leading to superior discrimination between benign and malignant lesions compared to 2D-based morphological characteristics (2).

Recent guidelines for lung nodule management recommend volumetric analysis over 2D assessment (3). This shift was largely driven by the Dutch–Belgian NELSON lung cancer screening trial, which employed volumetric analysis criteria and achieved a much lower false-positive rate than prior CT-based lung cancer screening trials (4, 5). Volumetric analysis combined with VDT analysis has emerged as an accurate predictor of malignancy (6).

While CT has been established as the gold standard for lung cancer screening, data on the potential of MRI as a radiation-free alternative are scarce (7). Although it has been shown that MRI reliably detects nodules >6 mm (8), in theory enabling MRI-based lung cancer screening, MRI based volumetric lung nodule assessment has only been evaluated *in vitro* thus far or in small human populations (9, 10). Delacoste et al. (9) conducted a pilot study demonstrating the feasibility of MR-based volumetric assessment, but the findings were limited to *in vitro* and *ex vivo* models. More recently, Darçot et al. (10) prospectively compared MRI and CT in the detection and volumetric assessment of lung nodules, reporting promising results but highlighting the need for further validation in larger cohorts. Therefore, the goal of this study was to assess the feasibility of in-vivo MRI-based lung nodule volumetry compared to CT, the reference standard for lung nodule assessment.

## Materials and methods

### Study participants

**FIGURE 1:**
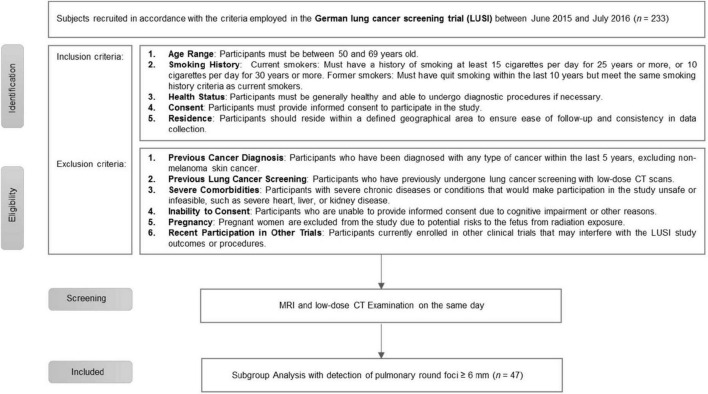
Flow diagram of study enrollment.

Patients from a prospective, single center, lung cancer screening program employing both CT and MRI were used for this subgroup analysis. A total of 233 participants were recruited following the criteria employed in the German lung cancer screening trial (LUSI; 11). Study enrollment is depicted in Figure 1. In the subgroup analysis, patients were included if round foci ≥6 mm were detected in CT during the initial screening round. Of the 233 participants, 23 met the inclusion criteria with a total of 47 pulmonary round foci ≥6 mm. This study was approved by the local ethics committee, patients gave written informed consent prior to inclusion in the study.

### Image acquisition

Patients underwent same-day MRI and low-dose CT (LDCT), regardless of whether pulmonary nodules were identified. LDCT was performed on a 128-slice spiral CT scanner (iCT, Philips Healthcare, Best, The Netherlands) in inspiratory breathhold with a reconstructed slice thickness of 1 mm and an increment of 0.6 mm.

MRI was performed on a 1.5 Tesla unit (Ingenia, Philips Healthcare Best, The Netherlands) in feet-first, arms-up technique using a phased array body coil. The employed MRI protocol has been previously described in detail (5). The scan protocol employs a transverse T2-weighted MultiVane XD (MVXD) imaging technique. The repetition time (TR) ranges from 950 to 1100 ms, and the echo time (TE) is set at 60 ms, with a flip angle (FA) of 90 degrees. The field of view (FOV) measures 400 mm, and the matrix size is 432 × 432 mm, providing detailed resolution. The slice thickness is maintained at 6 mm, in alignment with a previously validated protocol from our institution for two-dimensional nodule measurement (5). To enhance imaging efficiency, parallel imaging with SENSE (sensitivity-encoded) is utilized, and no partial Fourier technique is applied. Respiratory gating is employed to accommodate patient breathing, eliminating the need for breath-holding. The total acquisition time for this scan is 3 min and 18 s.

### Data analysis

Computed tomographic and MR images were reviewed by two experienced radiologists with 9 (R1) and 10 years (R2) of experience in chest imaging, respectively. The data sets were anonymized and randomly presented to the readers preventing direct inter-modality correlation. To further avoid bias, the readers first performed nodule analysis on MR images and after an interval of 2 weeks nodule analysis was performed on CT images.

Computed tomographic and MRI volumetric nodule assessment was performed with a computer-aided diagnosis system (CAD, IntelliSpace Portal DX Server, Philips Healthcare) which allows for both automated and semiautomated nodule volumetry.

**FIGURE 2:**
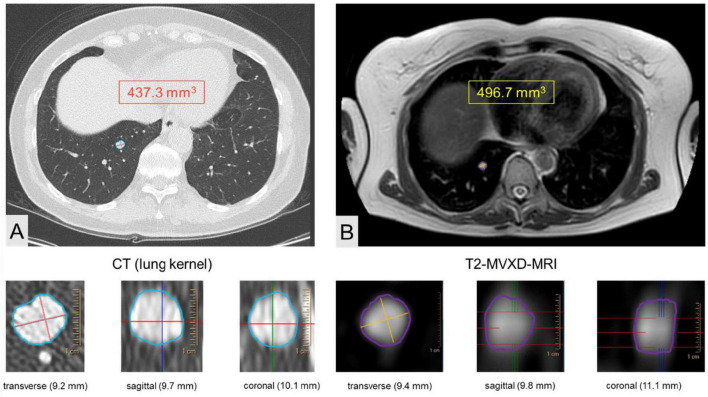
Volumetric assessment of a lung nodule in the right lower lobe using CT **(A)** and T2 weighted MVXD MRI **(B)** in a 56-year-old study patient.

For both MRI and CT semiautomated assessment was performed; after manual identification of the various nodules the software automatically contoured the nodule edges on each slice and calculated the nodule volume (Figure 2). If the computer-generated nodule borders appeared inaccurate, manual editing of computer-generated nodule outlines was performed.

### Statistical analysis

Statistical analysis was performed with SPSS 26 (IBM, Armonk, New York, USA). Subjects’ demographic were summarized descriptively. Continuous variables were expressed as means ± standard deviations. Regression analysis and Bland-Altmann analysis were conducted to assess correlation and intermodality agreement, between CT and MRI based lung nodule volumetry. The interclass correlation coefficient (ICC) was calculated to determine the inter/intra-reader reproducibility.

## Results

**FIGURE 3:**
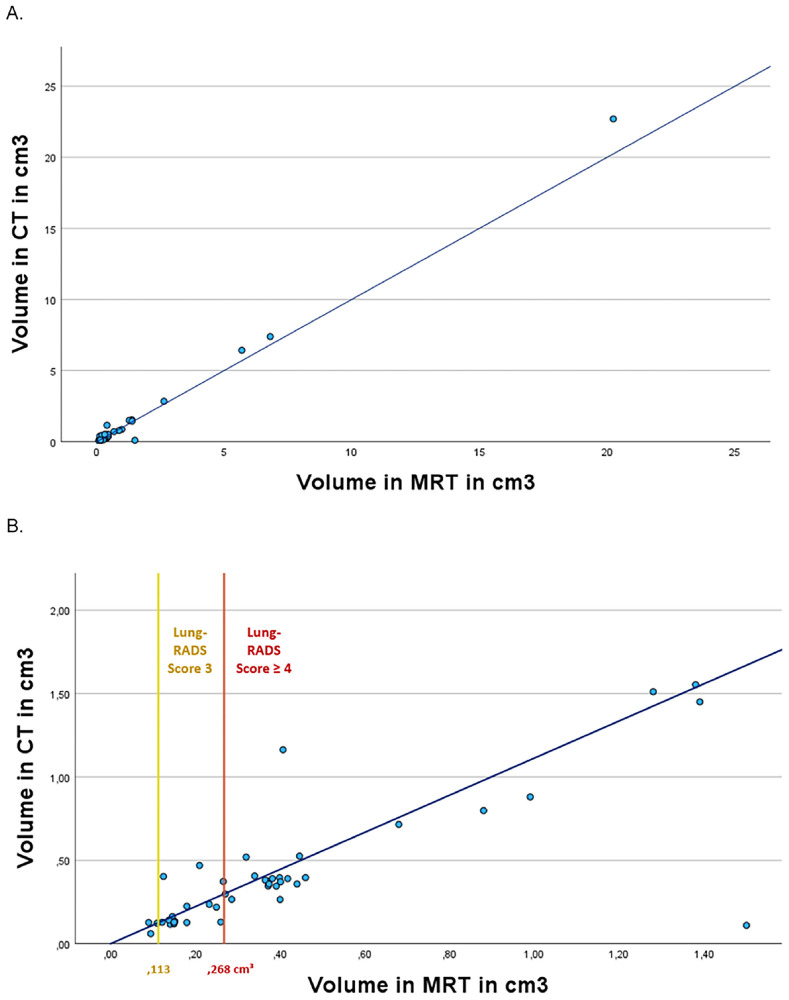
Regression analysis of CT-and MRI-based lung nodule volumetry. Correlation analysis between CT- and MRI-based lung nodule volumetric assessment for all nodules **(A)**; regression plot for nodules ≤1.5 cm^3^ **(B)**. The blue regression line represents the relationship between CT and MRI measurements. Additionally, Lung-RADS-based cutoff values have been integrated: category 3 (yellow; 0.113–0.268 cm^3^) and category 4 (orange; ≥0.268 cm^3^).

Based on LDCT as reference, mean nodule volume was 1.2197 ± 3.496 cm^3^, MRI-based mean nodule volume was 1.1343 ± 3.1204 cm^3^ (*p* = 0.203). Regression analysis revealed a linear relationship between the two modalities (*r*^2^ = 0.981; *p* < 0.001; Figure 3A); when considering nodules ≤2.5 cm^3^ (*n* = 44) a linear relationship was still evident (*r*^2^ = 0.755; *p* < 0.001) (Figure 3B).

**FIGURE 4:**
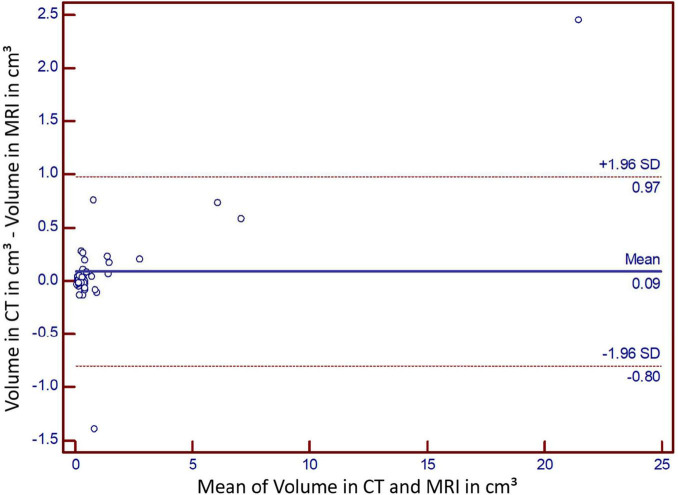
Bland-Altman analysis for intermodality comparison.

Bland Altman analysis revealed no systematic over- or underestimation of MRI in comparison to CT; mean difference: 0.12; standard deviation of differences: 3.496 (95% CI: 6.854 to −6.852) (Figure 4).

The intra-reader reproducibility assessment of CT based nodule volumetry resulted in an ICC of 0.9984 (95% CI: 0.9972 to 0.9991) for MRI based measurements ICC was 0.7737 (95% CI: 0.5142 to 0.9183). Inter-reader reproducibility revealed an ICC of 0.995 (95% CI: 0.993, 0.997) for CT and 0.7135 (95% CI: 0.4538 to 0.8581) for MRI-based measurements.

## Discussion

This study demonstrates that MRI-based volumetry of lung nodules larger than 6 mm is feasible and shows comparable precision to the current reference standard, CT.

Follow-up of lung nodules, including in lung cancer screening, is often excessive due to the need to monitor even low-risk nodules, leading to frequent imaging and potential overdiagnosis. This high frequency of follow-ups can expose patients to unnecessary radiation and stress, especially when nodules are benign or slow-growing. In high risk individual nodules up to 8 mm have less than 1% estimated malignancy risk, and those up to 15 mm in baseline (category 4A) have a 5%–15% risk (12). In a non-risk population, nodules smaller than 1 cm have an even lower malignancy risk (13). To avoid unnecessary radiation during follow-ups, MRI could serve as an alternative.

While MRI reliably detects lung nodules larger than 4−6 mm (14, 15), limited data exists on MRI-based volumetry beyond *in vitro* and initial *in vivo* testing. Ohno et al. evaluated only 2D nodule measurement in their study with 205 participants, without assessing volumetric measurements (16). Their findings demonstrated a high detection rate for lung nodules and feasibility for Lung-RADS classification, but volumetric evaluation was not performed. Volumetric assessment is standard practice in CT, as endorsed by national guidelines and the European position statement on lung cancer screening (3, 17). In this context, the research group led by Biederer further demonstrated that, compared to diameter measurements, volumetry results in significantly smaller interobserver variability, with advanced volumetric algorithms being independent of observer experience (18).

The current results indicate only minimal bias in artificial nodule volume measurements between MRI and CT, with a discrepancy of less than 10% across the volume range. High agreement is crucial when switching modalities for follow-up. This study also identified equivalent reproducibility between MRI and CT for pulmonary nodule volumetry, ensuring consistent results across different sessions and operators.

Appropriate MRI sequence selection has been addressed in several previous studies. The current volumetric analysis was performed using a T2 TSE sequence, a choice supported by existing literature, indicating that T2 sequences yield high nodule detection rates (14, 19). Additionally, newer ultra-short echo time (UTE) based MRI sequences have also shown promising results. These UTE sequences provide high detection rates and excellent volumetric accuracy.

Notably, the employed propeller technology enables reduction of motion artifacts and thus ensures precise measurements (14). Furthermore, the additional employment of a free breathing sequence allows for inclusion of patients unable to reliably hold their breath for longer periods, as typically required during standard imaging. This aspect is of particular importance in the context of individuals with an extensive history of smoking, where comorbidities affecting lung function are frequent (20, 21).

Recently, initial results were published regarding MRI based volumetric analysis of lung nodules (10, 22). In this study evaluating the effectiveness of UTE-based MRI sequences for lung nodule detection and volumetric assessment a high correlation was found between MRI and CT for nodule volumetry. UTE MRI provided high detection rates for nodules ≥4 mm and ≥6 mm, with an excellent concordance between CT and UTE-volumetry, albeit with a slight overestimation by MRI.

The current study, employing a more conservative MRI sequence, T2 TSE Propeller, also found comparable results for MRI in comparison to CT, although only nodules >6 mm were included. Smaller nodules were not included in the current analysis due to the restricted detection rate reported in previous T2 TSE based studies (23). However, nodules smaller than 6 mm typically have a low clinical relevance, therefore follow up will only seldomly be required. In comparison to UTE, the T2 TSE Propeller technique employed in this study offers significant advantages, such as the ability to perform acquisitions during free-breathing and a superior reduction of motion artifacts. These features make the sequence particularly suitable for patients with compromised lung function, enhancing its applicability in clinical settings.

Although MRI offers numerous potential advantages compared to the gold standard CT, it is unlikely to replace CT as the primary screening tool for lung cancer or for staging pulmonary metastasis. Nevertheless, MRI can be highly beneficial in certain scenarios, particularly for individuals sensitive to radiation exposure, such as young patients requiring frequent follow-ups. Additionally, MRI is advantageous when follow-up imaging of nodules larger than 4−6 mm is necessary and the goal is to avoid repeated CT scans.

MRI can be utilized effectively in follow-up imaging due to its ability to provide high-resolution images without the associated radiation risk of CT. This makes it an excellent choice for monitoring nodules over time, especially in patients who are at a higher risk of radiation-induced complications. For instance, young patients, who are more susceptible to the long-term effects of radiation, can benefit significantly from MRI’s non-ionizing imaging technology.

This study exhibits several limitations. First and foremost, the primary limitation is the relatively small sample size of analyzed lung nodules. While the measurements were diligently carried out by two experienced radiologists, each with 9–10 years of expertise, it is prudent to question whether comparable measurement results would be obtained if less experienced examiners were involved. To enhance the generalizability and reliability of the study’s findings, a larger and more diverse study population, as well as assessments by multiple readers, would be essential. Additionally, the volume measurements were executed utilizing a CAD system. It is crucial to acknowledge that different CAD systems may introduce variations in volume measurements, as evidenced by prior research (24, 25). Thus, it becomes imperative to exercise caution when extrapolating these findings to other CAD systems or methodologies, emphasizing the need for further investigation in this regard. Another noteworthy limitation lies in the absence of an exploration of inter-scan reproducibility in this *in vivo* study, which could be especially relevant for MRI scanners. The oversight of this aspect can potentially limit the reliability of the measurements over time, highlighting the need for future studies to comprehensively assess the technique’s repeatability. Furthermore, the study adhered to a protocol that exclusively included nodules larger than 6 mm. This decision aligns with the known limitation of MRI in detecting smaller lesions. However, it’s imperative to acknowledge that this study may not account for the full spectrum of lung nodules, particularly those with sizes below the 6 mm threshold. Additionally, subsolid nodules, which have distinct clinical significance, were not considered in the study, further narrowing the scope of its applicability.

## Conclusion

In conclusion, this study demonstrates the feasibility of MRI-based volumetric analysis for lung nodules ≥6 mm, with results comparable to CT. MRI offers a radiation-free alternative for lung cancer screening and follow-up, particularly for minimizing radiation exposure.

Future research should focus on validating MRI volumetry in larger, diverse populations and exploring its potential for early lung cancer detection and nodule classification using advanced AI algorithms. Multi-center studies and long-term follow-up are essential to further assess its clinical benefits.

## Data Availability

The raw data supporting the conclusions of this article will be made available by the authors, without undue reservation.
